# Supplementary material for: Persistent Cytotoxic Immune Signaling in Anti-VEGF–Treated Neovascular Age-Related Macular Degeneration
Source: medRxiv. 2026 Apr 10:2026.04.06.26350115. Preprint. [Version 1] doi: 10.64898/2026.04.06.26350115 (PMC13501240; doi:10.64898/2026.04.06.26350115)
Supplement: Supplementary file 1 [file NIHPP2026.04.06.26350115v1-supplement-4.pdf]

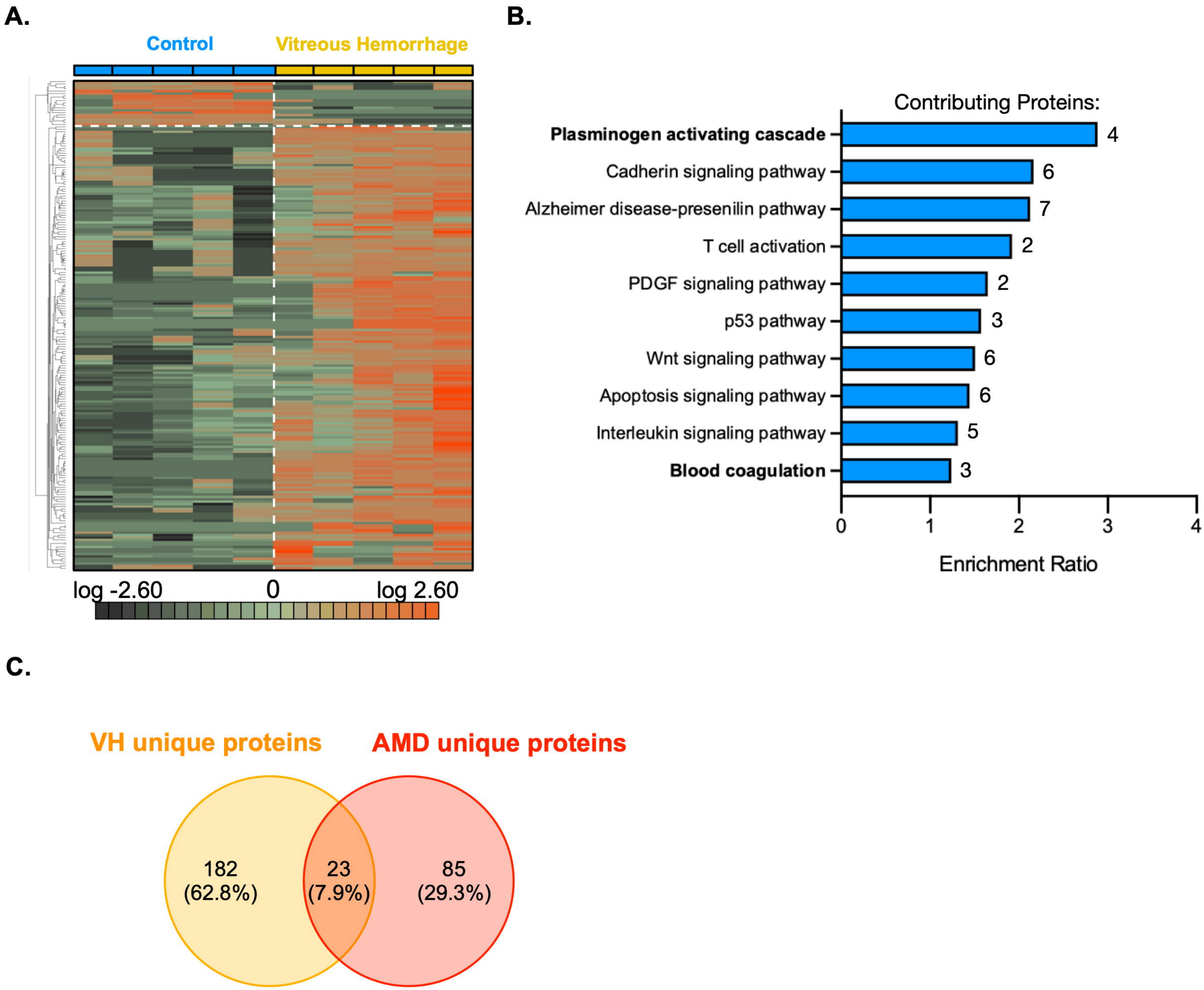

Supplemental Figure 1. Vitreous hemorrhage proteomic signal differs from AMD

A. Five vitreous hemorrhage (VH) and five comparative control vitreous biopsies were used for proteomic analysis as in Figure 1. Protein levels (pg/mL) from the multiplex ELISA assay were analyzed by 1-way ANOVA and differentially-expressed proteins ($p<0.05$) were represented as a heat map. 205 differentially expressed proteins were identified in total, with 19 down-regulated in VH and 186 upregulated. **B.** PANTHER pathway analysis was performed on this dataset, as in Figure 2, to identify the most active protein pathways in VH compared to controls. The number of genes contributing to the enrichment score from each gene set is listed to the right beside each bar. **C.** A Venn diagram comparison of differentially expressed proteins identified in VH and AMD. 182 unique VH proteins were identified and 85 unique AMD proteins were identified, while 23 shared proteins were found to be differentially expressed in both conditions.
